# Bone lesions of the tibia: Multimodal iconographic review and diagnostic algorithms, Part 1: Diagnostic algorithms, dysplasia and diaphyseal lesions

**DOI:** 10.1016/j.ejro.2025.100653

**Published:** 2025-05-02

**Authors:** Vincent Salmon, Pedro Augusto Gondim Teixeira, Alain Blum

**Affiliations:** Service d’Imagerie Guilloz, Hôpital Central, CHRU Nancy, Nancy 54000, France

**Keywords:** Tibia, Bone lesions, Dysplasia, Diaphysis

## Abstract

This article focuses on the analysis of bone lesions of the tibia, addressing the main diagnostic challenges and imaging strategies used to characterize them. It examines the different etiologies of tibial lesions, emphasizing the importance of a systematic approach to distinguishing tumoral from non-tumoral lesions, as well as from bone dysplasia. The article underlines the essential role of imaging, particularly radiography, CT, and MRI, in accurate lesion characterization. It also highlights typical clinical and radiological features that help guide diagnosis and management. The main aim is to provide radiologists with clear guidelines for improving the identification of bony lesions of the tibia. Part 1 of this 2-part article proposes simplified diagnostic algorithms and some illustrations of dysplasia and diaphyseal lesions of the tibia.

## Highlights

• Diagnostic algorithms streamline tibial lesion characterization via multimodal imaging.
• Systematic imaging helps distinguish tumoral and non-tumoral bone lesions of the tibia, but also dysplasia.
• Tibial dysplasia is identified through radiographic features and clinical associations.
• Different diaphyseal lesions are characterized by some imaging diagnostic features.
• Stress-related injuries and metabolic disorders can mimic tumor-like bone alterations.

## 1 Introduction

Tibial bone lesions are a common clinical occurrence. While many of these lesions are not specific to the tibia and may be found in other bones, some show a particular predilection for this long bone. They can present significant challenges for diagnosis due to the overlap between benign, malignant, and dysplastic lesions. Misinterpretation of these lesions can lead to inappropriate treatment and negatively impact patient outcomes.

This article presents a multimodal imaging review of tumoral, non-tumoral, and dysplastic lesions of the tibia. The primary focus is on diagnostic strategies that integrate clinical context and imaging characteristics.

This article is a narrative review without systematic approach. The literature was mainly chosen from recent imaging articles talking about bone lesions, especially concerning tibia.

In Part 1 of this 2-part article, we propose simplified diagnostic approaches according to the location of tibial lesions, followed by some illustrations of dysplasia and diaphyseal lesions.

The second part will focus on metaphyseal and epiphyseal lesions.

## 2 Diagnostic algorithms

**Fig. 1:**
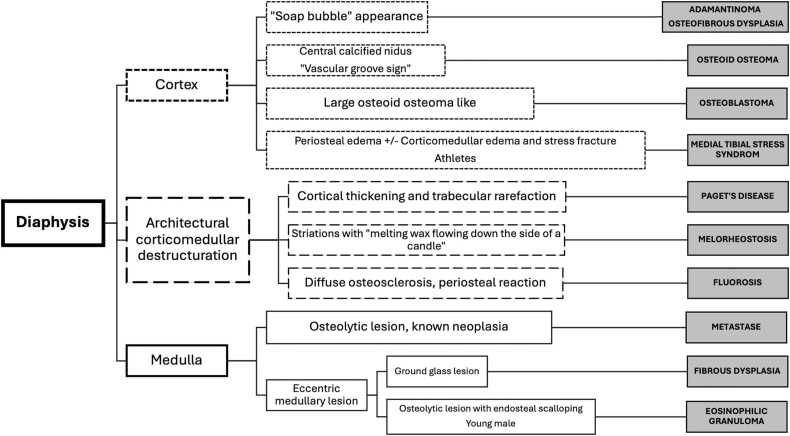
Algorithm for the diagnostic of osseous diaphyseal lesions of tibia.

**Fig. 2:**
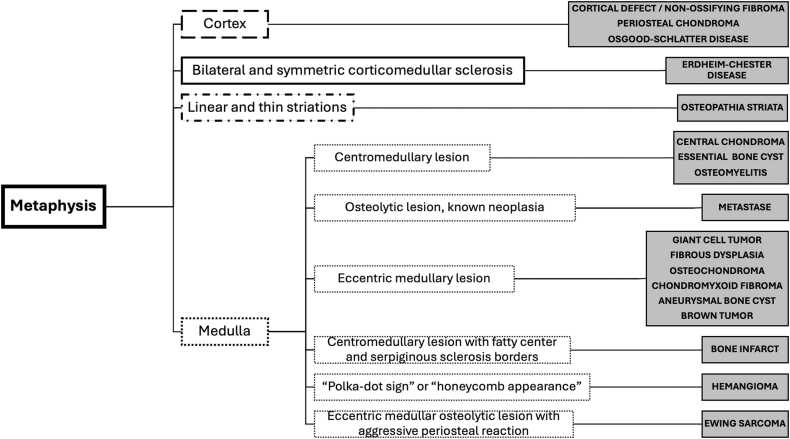
Algorithm for the diagnostic of osseous metaphyseal lesions of tibia.

**Fig. 3:**
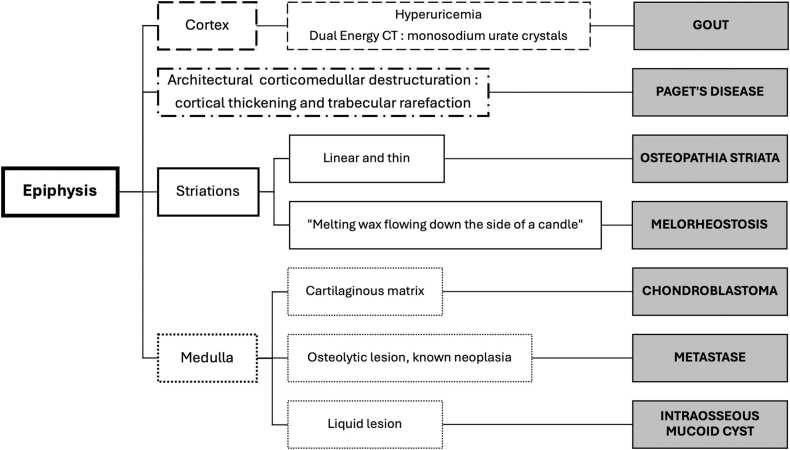
Algorithm for the diagnostic of osseous epiphyseal lesions of tibia.

To simplify the diagnostic process, this article introduces algorithms based on the anatomical location of tibial lesions (Fig. 1, Fig. 2, Fig. 3). Lesions are categorized by their position in the diaphysis, metaphysis, or epiphysis, with each category presenting distinct etiological considerations. The goal is to facilitate a structured and reproducible approach to tibial lesion assessment, aiding radiologists in distinguishing between various pathologies.

## 3 Dysplasia

Tibial dysplasia represents a rare but potentially severe condition, occurring in approximately one per million births [1]. While some cases arise sporadically, others are linked to syndromes characterized by multiple skeletal and visceral abnormalities, such as cardiovascular or genitourinary malformations.

### 3.1 Congenital tibial incurvation

Congenital tibial incurvation manifests as a curvature in the middle or distal third of the tibia, potentially affecting one or both limbs. Fibular curvature is frequently associated. The condition is clinically suspected in the presence of lower limb deformity. Radiographically, the tibia frequently shows corticomedullary abnormalities, including cortical thickening in the concavity of the deformity, cortical thinning at the apex of the convexity, trabecular osteocondensation and pseudocyst formation [2].

**Fig. 4:**
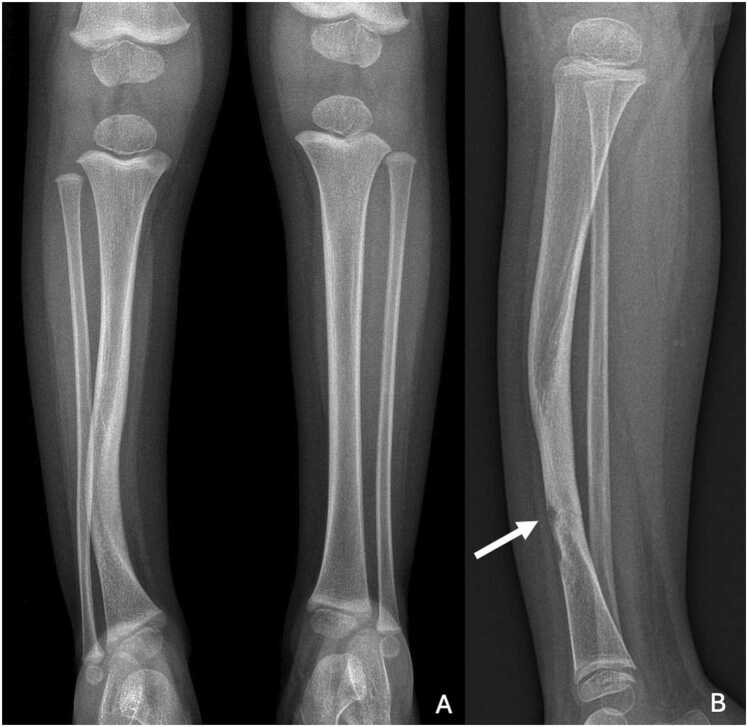
Right congenital tibial incurvation in two-year-old girl with a history of neurofibromatosis type 1, complicated by fracture. A. Face radiography with anterolateral convexity of the right tibia. B. Profile radiography performed a year later showing a fracture of the distal third of the tibia (white arrow).

Simply, tibial curvature can be categorized into three forms according to its orientation:

- Posteromedial convexity: generally associated with a favorable prognosis, often resolving within the first few years of life.
- Anterolateral convexity: this variant is linked to poorer outcomes, commonly associated with neurofibromatosis type 1, and may lead to pathological fractures or pseudarthrosis. Congenital pseudarthrosis of the tibia is rare in the general population, affecting between one in 140,000 and 250,000 births. However, it is more common in cases of neurofibromatosis type 1: the prevalence can be as high as four or five per cent [3] (Fig. 4). Diagnosis of pseudarthrosis is initially based on standard radiography. There are many pseudarthrosis classifications, the most widely used being Crawford's, which is mainly descriptive. It shows the stages of development of this pathology [4], [5], [6] (Table 1).

**Table 1:** Crawford’s classification in pseudarthrosis of tibia.

	**Type I**	**Type II**	**Type III**	**Type IV**
**Radiological features**	Anterior curvature Increased cortical density Narrow medullary canal	Anterior curvature Sclerotic and narrowed medullary canal	Anterior curvature Cyst or prefracture condition	Anterior curvature Full fracture with pseudarthrosis (often involving both tibia and fibula)
**MRI features of pseudarthrosis site**	Moderate hypersignal T2 Moderate hyposignal T1 Contrast enhancement Some adjacent soft-tissue abnormalities	T2 hypersignal T1 hyposignal Cortical thickening No adjacent soft-tissue abnormalities	T2 hypersignal T1 iso or hypersignal Thinning of bone cortex without disruption	T2 hypersignal T1 hyposignal Thinning and tapering bone, with bone disruption Contrast enhancement Thickening and enhancement of adjacent periosteum
**Diagrams (adapted from Crawford’s classification)**
- Anteromedial convexity: also associated with a poor prognosis, this form is often accompanied by fibular hemimelia. Bilateral and symmetrical tibial curvature and fibular hemimelia, associated with growth delay, can be part of Weismann-Netter-Stuhl syndrome.

When congenital tibial curvature is suspected as part of a syndromic presentation, further imaging should be conducted to assess for additional skeletal or visceral abnormalities, particularly in cases of neurofibromatosis type 1.

### 3.2 Tibial hemimelia

Tibial hemimelia, formerly known as internal longitudinal ectromelia, is a severe malformation, ranging from tibial hypoplasia to complete agenesis. It can be associated with femoral or fibular malformations. It may be unilateral or bilateral.

Diagnosis is often made antenatally via ultrasound. Clinically, the affected limb appears short and exhibits varus or flexion deformity.

Clément's classification is commonly used to distinguish four forms of tibial hemimelia, primarily based on the quality of the knee joint [7]:

- Type I: complete absence of the tibia, with a prominent and high-positioned fibula. This is the most common form.
- Type IIa: a proximal tibial cartilage model that later ossifies, with the fibula positioned lower than in type I.
- Type IIb: presence of an ossified proximal tibial metaphysis and a normally positioned fibula.
- Type III: distal tibial presence, with agenesis of the proximal two-thirds of the bone.
- Type IV: distal tibial hypoplasia with diastasis of the distal tibiofibular joint.

Assessment for tibial hemimelia usually begins with standard radiographs of both legs. Ultrasound or MRI may help visualize non-ossified tibial cartilage in type IIa cases. A thorough evaluation should be conducted to identify associated skeletal or visceral malformations, particularly in syndromic presentations.

### 3.3 Diaphyseal dysplasia

Diaphyseal dysplasia refers to sclerosis of the long bone diaphysis, particularly affecting the tibia. This rare condition is associated with two primary genetic disorders: Ribbing disease and Camurati-Engelmann disease. Both are very rare.

Some forty cases of Ribbing disease have been reported in literature [8]. In most cases, it affects tibia, bilaterally but asymmetrically in over 75 % of cases. Femur and humerus may also be affected. It is usually discovered around the age of 35, either by pain or by chance. There may be a progression to involvement of upper limb bones [8]. On standard radiographs, Ribbing disease presents with thickening of the endosteal and periosteal cortical layers in the diaphysis, narrowing the medullary canal without involving the metaphysis or epiphysis [9]. CT scan provides a better visualization of radiographic findings. MRI shows diaphyseal bone edema without extension to soft tissues. Bone scintigraphy shows diaphyseal hyperfixation.

Camurati-Engelmann disease is the main differential diagnosis for Ribbing disease. Its frequency is estimated at 1/1,000,000, with around 200 cases reported [10], [11]. Diagnosis is usually made in adolescence, in presence of pain, but can be difficult due to a gait that suggests a muscular origin. Diaphyseal tibial involvement is bilateral and symmetrical but differs from Ribbing disease in its metaphyseal extension and possible involvement of vertebrae and skull. Standard radiography and CT scan reveal fusiform thickening of cortical endosteal and periosteal layers, responsible for narrowing of medullary canal [12]. Bone scintigraphy shows hyperfixation but may be normal.

### 3.4 Blount's disease

Blount's disease, or tibia vara, is an acquired growth disorder of proximal tibia, at the level of epiphysis and plate growth, responsible for a multiplanar deformity of tibia, particularly in varus [13]. It is an uncommon pathology, classified into different types according to age of the child:

- Infantile: between two and five years old.
- Juvenile: between five and ten years old.
- Adolescent: after ten years old.

Infantile form is the most frequent, particularly in obese boys of Afro-American origin who acquired walking at an early age. This is the most severe form, although it does not usually cause pain initially. It is bilateral in 50 % of cases. Adolescent form is the rarest and is unilateral in almost 90 % of cases.

On standard front radiography, a genu varum is measured by the metadiaphyseal angle (or Levine-Drennan angle), which defines Blount's disease when it is greater than 11°. This angle is formed between a line perpendicular to the long axis of tibia and a line tangent to proximal tibial metaphysis. Morphological changes of proximal tibial epiphysis and growth plate are present. There is no significant abnormality of the more distal portion of tibia, nor of distal end of femur in infantile form. Langenskiold's classification of proximal tibial deformity into six grades according to patient's age [14].

CT scan, with three-dimensional reconstructions, can play a role in surgical planning [15].

MRI is useful for visualizing whether plate growth is closed. It is also an aid to surgical or non-surgical management.

## 4 Diaphyseal lesions

### 4.1 Adamantinoma, osteofibrous dysplasia and differentiated adamantinoma

Adamantinoma, osteofibrous dysplasia (OFD) and differentiated adamantinoma are part of the same lesion spectrum [16].

Adamantinoma is a locally aggressive, low-grade malignant tumor. It is rare, accounting for less than 1 % of primary malignant bone tumors, and typically affects young children and adults with an average onset age of 30 years [17]. The tibia is the preferred site of adamantinoma in 80–90 % of cases, sometimes accompanied by fibular involvement. The tumor primarily affects the tibial diaphysis, commonly in the anterior cortex. Pain is the primary symptom, though leg deformities may occasionally occur. Complications such as pathological fractures are rare. Local recurrences and lung metastases can occur on a long duration [18].

Radiographically, adamantinoma presents as a large, multiloculated lesion with lacunae and peripheral sclerosis, producing a "soap-bubble" appearance. It usually exceeds 5 cm in size, elongated along the long diaphyseal axis. Cortical destruction, while suggestive of the diagnosis, is not always present, though "moth-eaten" borders are typically seen.

On CT, adamantinoma is easier to characterize. Cortical destruction is also simpler to identify. In addition, this imaging modality can be used to search for satellite lesions on the same bone, which is not uncommon.

On MRI, adamantinoma appears isointense to muscle on T1-weighted images and hyperintense on T2-weighted sequences, with a homogeneous or occasionally cystic appearance. Involvement of osteomedullar cavity is variable. Intense contrast enhancement is typical, but soft-tissue extension is rare [19].

Bone scintigraphy usually shows tumor hyperfixation.

Differentiated or juvenile adamantinoma or osteofibrous dysplasia-like adamantinoma (OFD-LA) are different names for another form of adamantinoma. This bone tumor is benign. Classical adamantinoma is generally characterized by a mixture of epithelial and osteofibrous components in varying proportions. Differentiated adamantinoma consists of predominantly osteofibrous tissue, in which small groups of epithelial cells are detected only by careful investigation or immunohistochemistry [20].

Imaging appearance of differentiated adamantinoma is very similar to that of osteofibrous dysplasia.

Osteofibrous dysplasia, or ossifying fibroma, is a very rare benign bone tumor, accounting for only 0.2 % of all primary bone tumors. It mainly affects children and young adults under the age of 20. The tibia is the most common site, representing 75 % of cases. The lesion typically occurs in the diaphysis and often presents with a visible swelling or abnormal curvature of the tibia. Pain is rare unless a pathological fracture occurs.

On standard radiographs, osteofibrous dysplasia appears as a multiloculated osteolytic lesion with sclerotic margins, predominantly affecting the anterior cortex of the tibial diaphysis. The cortex may be expanded with occasional areas of thinning. In 50 % of cases, an involvement of the osteomedullary cavity is seen. "Moth-eaten" borders are less frequently seen than in adamantinoma. The lesion typically extends along the long axis of the diaphysis for about 3 cm.

On CT, characteristics of osteofibrous dysplasia are more easily recognized. It can sometimes present a ground-glass appearance.

On MRI, lesion is isosignal T1 and more generally isosignal T2, separated by hyposignal T1 and T2 fibrous septa. Contrast enhancement is heterogeneous. In 50 % of cases, there is osteomedullary and adjacent soft-tissue edema.

Bone scintigraphy shows hyperfixation of the lesion.

Histologically, osteofibrous dysplasia corresponds to scattered epithelial cells recognized by immunohistochemistry. Adamantinoma is made up of epithelial cells forming small nests recognized by hematoxylin and eosin staining [20], [21].

**Fig. 5:**
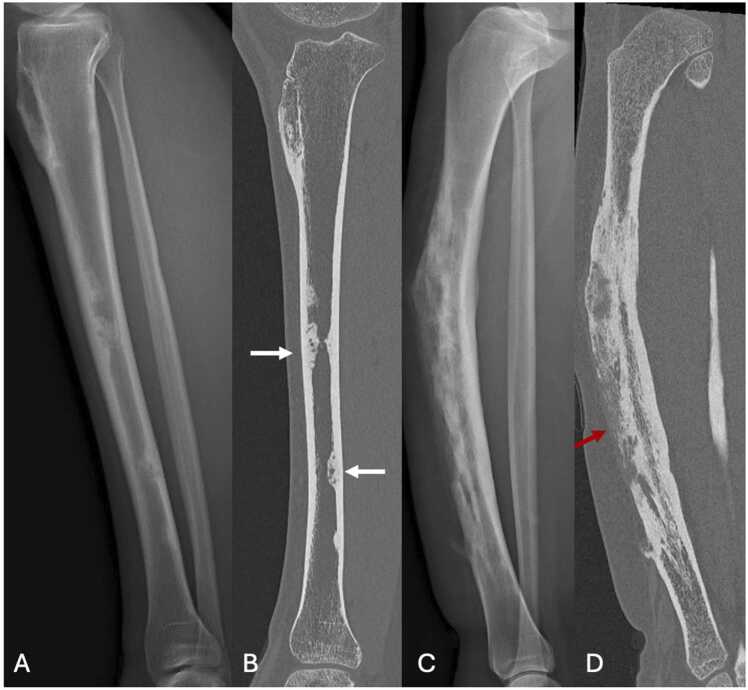
A,B. Plurifocal osteofibrous dysplasia of the left tibia in a 19-year-old young woman. C,D. Adamantinoma of the right tibia in a 14-year-old girl (different patient). A. Profile radiography showing plurifocal sclerotic lesions of diaphyseal cortical of the tibia. B. UHR CT in sagittal plane highlighting those sclerotic lesions with central hypodense areas, no cortical destruction and endosteal extension along the long axis of the diaphysis (white arrow). C. Profile radiography showing a large ill-defined lesion of the middle diaphysis of the tibia, with cortical thickening and multiple lacunae of the anterior cortical bone producing a « soap-bubble » appearance. Note the curvature of the tibia. D. UHR CT in sagittal plane highlighting cortical destruction of the anterior cortical bone (red arrow) and involvement of the medullar cavity.

Table 2 resumes differences between adamantinoma, osteofibrous dysplasia and differentiated adamantinoma. The distinction in imaging remains sometimes difficult to make. Fig. 5 shows plurifocal osteofibrous dysplasia and adamantinoma on two different patients.

**Table 2:** Differences between adamantinoma, osteofibrous dysplasia and differentiated adamantinoma.

**Features**	**Adamantinoma**	**Osteofibrous dysplasia (OFD)**	**Differentiated adamantinoma**
**Histopathology**	Mixture of epithelial and osteofibrous components in variable proportions	Scattered epithelial cells	Predominantly osteofibrous tissue, with small groups of epithelial cells
**Age**	Children and young adults	Children and young adults	Children and young adults
**Site**	Tibial diaphysis (90 %) Anterior cortex + /-medullar involvement	Tibial diaphysis (75 %), fibula Anterior cortex + /- medullar involvement	Tibial diaphysis Anterior cortex
**Size**	6.5–26 cm	4–8.5 cm	2–13 cm
**Conventional radiography CT scan**	Polygeodic lesion, with multiple lacunae and peripheral sclerosis "Soap-bubble" appearance Moth-eaten borders more frequent Periosteal reaction	Polygeodic lesion, with multiple lacunae and peripheral sclerosis Sometimes, ground glass appearance and moth-eaten borders Periosteal reaction	Similar to OFD
**MRI**	Intermediate signal intensity on T1w High signal intensity on T2w (homogeneous > heterogeneous) High and homogeneous contrast enhancement	Intermediate signal intensity on T1w Intermediate (rarely high) signal intensity on T2w (heterogenous > homogeneous) High and heterogeneous contrast enhancement	Similar to OFD
**Bone scintigraphy**	Tumor uptake	Tumor uptake	Tumor uptake
**Behavior**	Locally aggressive	Benign	Relatively benign

### 4.2 Osteoid osteoma

Osteoid osteoma is a relatively common benign bone tumor, accounting for around 12 % of all benign bone tumors [22]. It tends to affect young patients, between the ages of seven and 25, and rarely after the age of 30. Boys are affected two to three times more often than girls. Typically, this lesion is responsible for localized, inflammatory, insomniac pain, relieved by aspirin.

Osteoid osteoma commonly occurs in the long bones, particularly in the tibia and femur, where it accounts for almost 60 % of cases. It mainly affects the cortical bone at the diaphyseal level, at the junction with the metaphysis. More rarely, it can be found in the medullary or subperiosteal regions.

On standard radiographs, lesion appears as an oval lacuna, less than a centimeter in size [23]. Generally, there is peripheral sclerosis and sometimes central calcification corresponding to the classically described nidus.

**Fig. 6:**
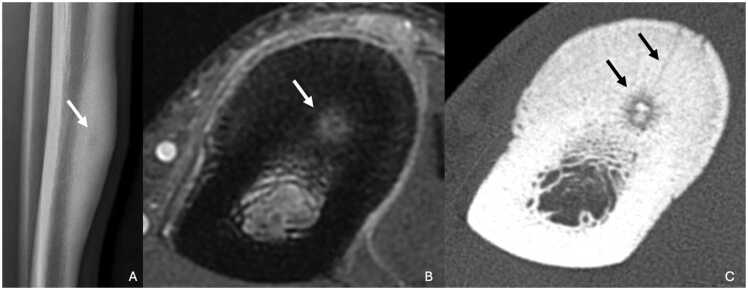
Osteoid osteoma of the diaphysis of the left tibia in a 16-year-old young man. A. Profile radiography showing thickening of the middle third of the tibial cortex with a central rounded clearness (white arrow). B. T1-weighted fat-suppressed contrast-enhanced MR image in axial plane showing a nodular enhancement of the anterior tibial cortex (white arrow) with periosteal enhancement. C. UHR CT in axial plane highlighting serpiginous low-density grooves radiating from periosteal surface to the central calcified nidus (medusa head sign, black arrows).

CT scan is the key imaging modality for diagnosing osteoid osteoma. CT scan more easily shows the cortical lacunar lesion and, above all, the central nidus. The vascular groove sign, corresponding to multiple peripheral serpiginous vessels, is in favor of osteoid osteoma [24]. Ultra-high resolution (UHR) scan can clearly highlight these vessels, as well as the afferent arteriole that penetrates the nidus [25] (Fig. 6). Perfusion CT may show early contrast enhancement followed by rapid washout, consistent with the hypervascular nature of the lesion. CT also plays a crucial role in guiding percutaneous treatment, especially radiofrequency ablation.

On MRI, the central nidus is typically hypointense on all sequences if calcified, but it may show intermediate or increased T2 signal intensity in non-calcified cases. The surrounding stromal tissue usually appears hyperintense on T2-weighted images and exhibits contrast enhancement. Inflammatory changes in the bone marrow and adjacent soft tissues can cause significant edema, potentially mimicking malignant or infectious processes.

Bone scintigraphy typically shows a characteristic "double density sign," with intense central uptake reflecting the nidus and less pronounced peripheral uptake corresponding to reactive sclerosis [26].

The differential diagnosis with a stress fracture may sometimes arise.

### 4.3 Osteoblastoma

Osteoblastoma is a rare benign bone tumor, comprising less than 1 % of all primary bone tumors.

Histologically, it presents similar characteristics to osteoid osteoma but it is not “a large osteoid osteoma”. It occurs preferentially in male children and young adults under the age of 30 [22], [27]. This lesion manifests as inflammatory pain, predominantly nocturnal, partially relieved by aspirin. In the tibia, osteoblastoma mainly affects the diaphysis, though the metaphysis can also be involved. The lesion can be located in the medullary, cortical, or periosteal areas. Osteoblastoma is often larger than osteoid osteoma, measuring between 2 and 10 cm.

On standard radiographs, osteoblastoma presents as a fusiform radiolucent lesion. Peripheral sclerosis and cortical thinning may be present. A central calcified nidus is strongly suggestive of the diagnosis. A periosteal reaction may also be present.

CT scans provide clearer delineation of the nidus and any intra-lesional calcifications.

MRI typically shows the lesion as hypointense or isointense on T1-weighted images and hyperintense or isointense on T2-weighted images [23]. Peripheral osteosclerosis may appear as hypointense on both T1 and T2 sequences. The lesion, along with adjacent soft tissues, often demonstrates contrast enhancement, a phenomenon known as the "flare phenomenon," which may lead to a false suspicion of more aggressive pathology [28].

On bone scintigraphy, there is intense aspecific hyperfixation.

### 4.4 Medial tibial stress syndrome

Periostitis and stress fracture are part of a lesion continuum within a broader pathological spectrum: "Medial Tibial Stress Syndrome" (MTSS). These injuries are caused by chronic repetitive stress on the tibia, particularly involving the Sharpey fibers. MTSS is most frequently seen in athletes, particularly runners, and accounts for 13–20 % of cases. There is a slight female predominance, particularly in women with amenorrhea [29].

Initially, MTSS may present as subtle pain with a mechanical rhythm that worsens with increased activity. If stress persists, the pain becomes constant, and subcutaneous swelling with periosteal reaction may develop. Although MTSS is primarily diagnosed clinically, imaging is often required to rule out fracture-like complications.

MTSS most commonly affects the cortical bone along the posteromedial border of the tibial diaphysis, especially in the distal two-thirds of the bone. Fractures of the anteromedial tibia are associated with a poorer prognosis.

Radiographic findings are often absent in the early stages of MTSS. Signs such as cortical thickening or a visible fracture line may not appear until 10 days after symptom onset and can still be absent in up to 50 % of cases [30].

Ultrasound can be useful in demonstrating cortical interruption and periosteal reaction.

**Fig. 7:**
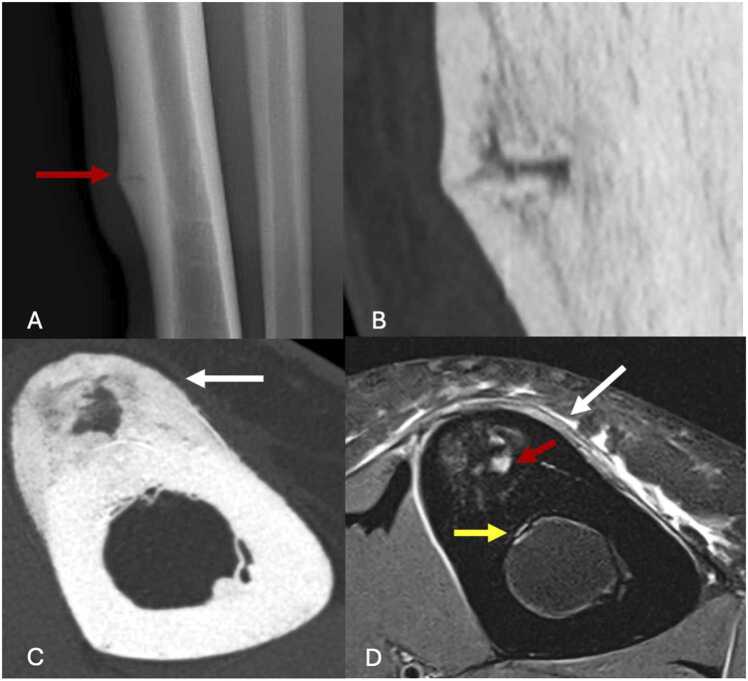
Stress fracture of the diaphysis of right tibia of a 15-year-old female adolescent playing volleyball at a high level with pain and swelling in her right leg for several months. A, Initial profile radiography, performed four months after onset of symptoms, showing cortical thickening of the distal-third of tibial diaphysis with discrete fracture line (red arrow). B, CT image in the sagittal plane highlighting the fracture line. C, CT image in the axial plane, performed with a Ultra-High Resolution CT scan, showing intense periosteal reaction and cortical thickening (white arrow). D, 2-month follow-up proton density-weighted fat-suppressed MR image in the axial plane showing periosteal (white arrow) and osteomedullar edema (yellow arrow) with fracture line (red arrow) (grade 4b of Fredericson’s classification).

A CT scan may show the fracture line, rarefaction of bone cortex and periosteal reaction (Fig. 7). CT scan can sometimes be misleading if performed too early.

MRI allows earlier detection of MTSS. Fredericson's classification was the first to propose a distinction into several grades [31], [32]:

- Grade 1: isolated periosteal edema, without medullary edema.
- Grade 2: periosteal and osteomedullar edema visible only on T2-weighted sequence.
- Grade 3: periosteal and osteomedullar edema visible on T2- and T1-weighted

- sequences.
- Grade 4a: periosteal edema, plurifocal cortical signal anomalies and osteomedullar edema visible on T2- and T1-weighted sequences.
- Grade 4b: periosteal edema, cortical fracture line and osteomedullar edema visible on T2- and T1-weighted sequences.

Other classification systems, developed by Arendt and Nattiv, also exist, with slight variations from Fredericson's classification [33], [34].

MRI helps eliminate misleading differential diagnoses in other imaging modalities. It serves to guide rest time until recovery.

Bone scintigraphy is a good tool for the detection of MTSS, but provides less information than MRI in the study of lesions, particularly in gradation [35]. In case of periostitis, hyperfixation generally extends over more than five cm, whereas it is between one and three cm in case of a stress fracture.

### 4.5 Paget's disease

Paget’s disease, also known as osteitis deformans, is a chronic bone disorder that results in abnormal and excessive bone remodeling. While the exact cause is uncertain, a combination of genetic and environmental factors is suspected. Paget’s disease primarily affects Caucasians, with higher prevalence in Western Europe and Australia, while it is rare in Asia and Africa [36]. Historically, its prevalence was around 5 % in Great Britain and 2 % in France during the 1970s. Its incidence has been decreasing over the last few decades [37]. Paget’s disease typically affects individuals over 55, with a slight male predominance.

It is the second most common metabolic bone disease after osteoporosis, progressing through three distinct phases: the lytic phase (characterized by high osteoclastic activity), the active phase (marked by intense osteoblastic repair), and the inactive phase (involving a gradual decrease in both osteoclastic and osteoblastic activity). The disease primarily affects the lumbosacral spine and pelvis, but long bones such as the tibia are also commonly involved. It begins at the epiphysis and slowly extends toward the diaphysis, sparing the joints unless ankylosis occurs.

Paget’s disease is often an incidental finding on imaging since most patients are asymptomatic. When symptoms are present, they typically include bone pain, skeletal deformities (especially in long bones), or complications such as fractures. Blood tests may reveal elevated serum alkaline phosphatase levels, indicative of increased bone turnover, though calcium levels are usually normal unless a fracture is present.

On standard radiographs, tibial involvement typically begins with cortical resorption at the epiphysis, progressing toward the diaphysis in a characteristic “blade of grass” or “flame-shaped” pattern. There is a clear demarcation from healthy bone. In some cases, resorption may start directly in the diaphysis [38]. In the osteoblastic phase, cortical thickening, bone trabeculation and widening of the bone become evident [39]. In the late phase, the resorption front disappears. The bone may take on a "saber-shaped" deformity [40].

**Fig. 8:**
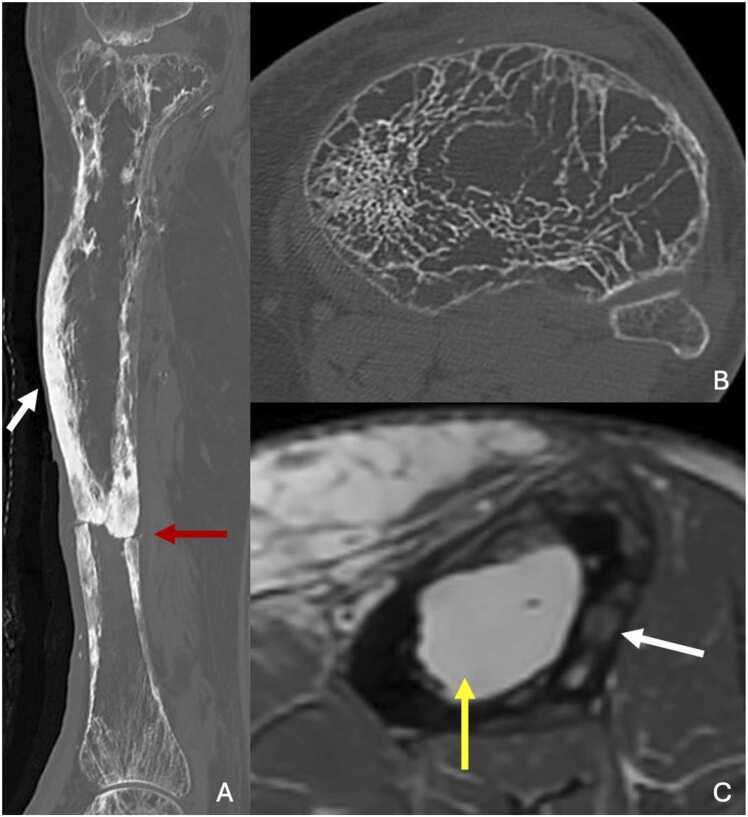
A. Paget’s disease of the right tibia in a 85-year-old-man. B,C. Paget’s disease of the left tibia in a 74-year-old-man (different patient). A. CT image in the sagittal plane showing a pathological fracture (red arrow) in a « saber-shaped » deformity of the tibia. Note the thickening of the anterior cortex (white arrow). B. CT image in the axial plane showing rarefaction and thickening of trabeculations of the epiphysis of the tibia. C. T1-weighted MR image in the axial plane showing a pronounced fatty signal of the medullar cavity (yellow arrow), cortical areas of resorption in hyposignal (white arrow).

CT provides further detail, revealing cortical thickening, dedifferentiation of the corticomedullary junction, thickening of the trabeculae and widening of the bone (Fig. 8).

MRI findings vary depending on the disease stage. In the osteoclastic stage, areas of cortical resorption are T2 hypersignal, T1 hyposignal and intensely enhanced. Adjacent soft tissue edema may be present. In osteoblastic stage, T1 and T2 signal abnormalities become more heterogeneous, as a fatty signal develops. This fatty signal is more pronounced than that of non- pathological osteomedullary fat. In the late phase, within fatty signal, there may be T1 and T2 hyposignals corresponding to osteocondensed areas.

Bone scintigraphy typically shows increased radiotracer uptake in all phases of Paget’s disease. Uptake may be less pronounced in the late phase.

Sarcomatous degeneration is a rare but serious complication, especially in polyostotic forms.

### 4.6 Melorheostosis

Melorheostosis, or Leri’s disease, is a rare, benign condition characterized by linear cortical hyperostosis and sclerosis of the medullary bone, most commonly affecting the lower limbs. This non-hereditary disorder typically affects bones along a single limb, unilaterally, and follows the path of sclerotomes [41]. Melorheostosis is caused by different somatic mutations such as MAP2K1, SMAD3, KRAS or LEMD3 [42]. In some cases, it may extend into adjacent joints, leading to soft tissue calcifications or ossifications that follow the myotomes and dermatomes.

Melorheostosis has an incidence of approximately 0.9 per million people. The gender distribution is roughly equal. Most cases are diagnosed in childhood, when patients present with pain, swelling, or limited joint mobility. Growth disorders, with unequal length of lower limbs, are common.

**Fig. 9:**
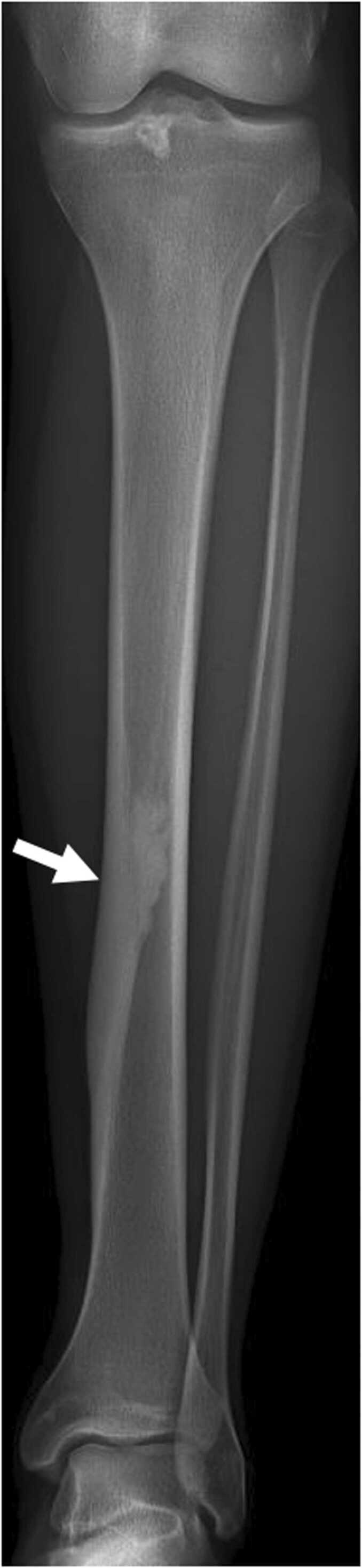
Melorheostosis in a 20-year-old young man with swelling of medial side of the left leg. Face radiography of the left leg showing endostotic hyperostosis of the middle-third of tibial diaphysis giving “melting wax flowing down the side of a candle” appearance (white arrow).

On standard radiographs, melorheostosis typically appears as irregular cortical hyperostosis of the long bones, sometimes accompanied by endostotic hyperostosis that obliterates the medullary canal. The radiological appearance is often described as "wax flowing down the side of a candle" [43]. In some cases, the disease may present as linear striations, mimicking osteopathia striata [44] (Fig. 9). Soft-tissue ossifications are usually para-articular.

CT scans provide better characterization of the hyperostosis, showing a more detailed view of cortical and medullary involvement. Soft tissues are often affected but remain unattached to the adjacent bone.

On MRI, the hyperostotic areas appear hypointense on both T1- and T2-weighted sequences. Soft-tissue masses show variable signal intensity depending on the degree of ossification and fat content.

Bone scintigraphy typically reveals intense radiotracer uptake in areas of pain [45].

### 4.7 Fluorosis and related disorders

Fluorosis is a chronic metabolic bone disease linked to ingestion of large quantities of fluoride. Ingestion of excess fluoride can occur through water and food in certain regions of the world where high fluoride levels constitute a public health issue, such as Asia (particularly India) and Africa [46]. Fluorosis is rare in Western Europe and North America, but cases have been described following excessive inhalation of difluoroethane or long-term use of antifungal agents such as voriconazole. Development of the disease is usually slow, taking several years, but is much more rapid in cases of contamination by difluoroethane or voriconazole.

Men are more frequently affected. Symptoms generally include bone or joint pain. Diagnosis often occurring in young adults around age 35 [47]. Dental fluorosis, characterized by white or yellow spots on the enamel, can be an early indicator of the disease. In cases of fluorosis, serum alkaline phosphatase and fluoride levels in blood and urine are elevated. Fluoride can also be detected in bone biopsy samples.

Clinical context, geographical origin and list of treatments taken are important in diagnostic reasoning.

On standard radiographs, diagnosis is suspected by diffuse osteosclerosis, calcification of tendons, ligaments and interosseous membranes, and vertebral osteophytes. In long bones, such as the tibia, there is thickening of bone trabeculae and cortex responsible for diaphyseal widening, associated with periosteal reactions.

**Fig. 10:**
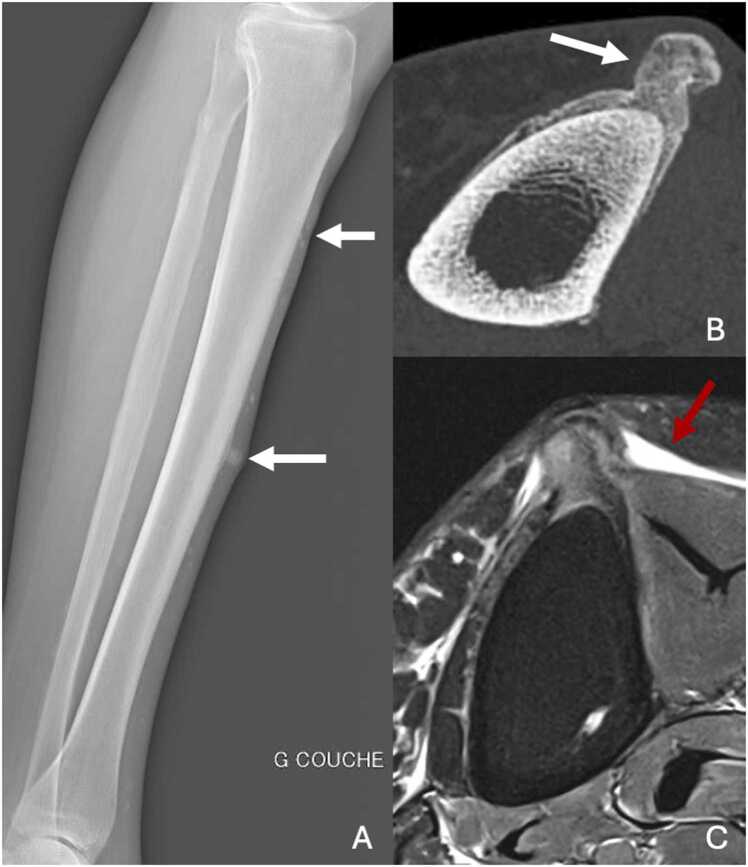
Voriconazole-induced periostitis in a 44-year-old woman with history of chronic end-stage renal disease treated by renal transplantation and invasive aspergillosis. A. Profile radiography of the left leg showing some plurifocal nodular calcifications (white arrows). B. CT image in the axial plane showing pronounced periosteal reaction with nodular aspect at the anterior edge of tibia (white arrow). C. Proton density-weighted fat-suppressed MR image in the axial plane showing hypersignal of periosteal reaction associated with adjacent neobursitis (red arrow).

In voriconazole-induced periostitis, periosteal reactions are smooth or nodular, multifocal and asymmetrical [48] (Fig. 10).

CT scans show coarse trabeculae and confirm the radiographic findings.

On MRI, there is a T1 and T2 hyposignal associated with osteosclerosis. Periostitis is isosignal T1 and hypersignal T2.

Bone scintigraphy shows intense bone hyperfixation of peripheral and axial skeleton, in favor of extensive bone remodeling.

### 4.8 Eosinophilic granuloma

Eosinophilic granuloma is a benign bone proliferation of Langerhans cells and is the bone manifestation of Langheransian histiocytosis (histiocytosis X). Histiocytosis X is a relatively rare condition, affecting less than 1 % of bone lesions. It affects young patients, less than 20 years of age in almost three-quarters of cases. There is a predominance of males [49].

In view of clinical and radiological polymorphism of histiocytosis X, we have chosen to focus only on radiological presentation of eosinophilic granuloma. The preferred location of eosinophilic granuloma is the cranial vault, but it can also affect long bones such as the tibia, where it occurs in about one-third of cases [50]. The disease often manifests as a solitary lesion (monostotic), though multiple bone lesions (polyostotic) are possible. The typical lesion is 4–6 cm in size. It primarily involves the diaphysis, with rare involvement of the epiphysis. Clinically, eosinophilic granuloma can cause pain, swelling and hyperthermia. It may lead to complications such as pathological fractures, though it is occasionally an incidental finding.

On standard radiographs, eosinophilic granuloma presents as a well-defined osteolytic lesion centered in the bone medulla and extending toward the cortex, causing endosteal scalloping [51]. Older lesions may show peripheral sclerosis [50]. A periosteal reaction may also occur.

CT scans provide better characterization of cortical involvement and soft-tissue extension in cases of aggressive osteolysis.

MRI can detect lesions not visible on other imaging modalities. Eosinophilic granuloma typically show a heterogeneous hyperintense signal on T2-weighted images and moderate hypointense signal on T1-weighted images. Significant adjacent bone marrow, periosteal, and soft tissue edema are often present in cases where the lesion penetrates the cortex [52]. Contrast-enhanced imaging reveals intense enhancement in both the lesion and surrounding areas. As the lesion heals, the degree of T2 hyperintensity and perilesional inflammation decreases.

Bone scintigraphy is of limited value, as only 50 % of lesions are hyperfixating. However, ^18^FDG PET scan is more sensitive for detecting asymptomatic lesions.

### 4.9 Intraosseous varicose veins

Intraosseous varicose veins are an extremely rare cause of venous drainage anomalies in the lower limbs. They are often occurring in the context of chronic venous insufficiency or post-phlebitic syndrome associated with varicose veins [53]. These lesions can appear at any age.

Patients typically present with chronic tibial pain. In most cases, involvement is bilateral and symmetrical. When a unilateral pathology interrupts venous return (e.g., a fracture), the lesion may be unilateral [54]. The abnormality is typically centered on the anterior surface of the diaphyseal cortex of the tibia.

On standard radiographs, there is an oval or serpiginous osteolytic lesion, depending on incidence, with slightly sclerotic contours [55]. Chronic venous insufficiency is also responsible for a periosteal reaction and cortical thickening. Phleboliths and edema of peri-tibial soft tissues contribute to diagnosis. Bone demineralization may also occur.

**Fig. 11:**
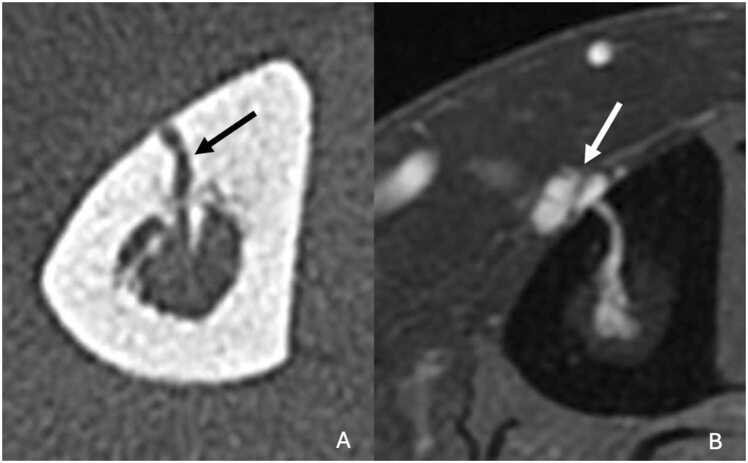
Intraosseous varicose vein in a 64-year-old woman with a history of surgery for bilateral varicose veins of lower limbs, presenting chronic pain in the left leg for several years. A. CT image in the axial plane highlighting this serpiginous vessel passing through the tibial cortex (black arrow). B. T1-weighted fat-suppressed and contrast enhanced MR image in the axial plane showing this dilated intraosseous vessel communicating with varicose vein touching the tibial cortex (white arrow). Interventional treatment by sclerotherapy has reduced symptoms.

CT scans are effective in confirming the presence of intraosseous venous dilation and provide a clearer view of cortical and periosteal changes linked to venous insufficiency (Fig. 11). A perfusion CT scan is useful for demonstrating progressive venous filling.

Doppler ultrasonography is used to analyze the direction of blood flow [56].

MRI is valuable for detecting intraosseous venous dilatation, which appears as T2 hyperintensity. Additional signs of venous insufficiency, such as periosteal and soft tissue changes, are also evident on MRI [57].

Interventional radiology treatment with sclerotherapy is part of therapeutic panel.

### 4.10 Metastases

Metastases are by far the most common bone tumors. In almost 80 % of cases, they originate from breast, prostate, lung, kidney and thyroid cancers. Breast cancer in women and prostate cancer in men are the most common primary cancers to give rise to distant bone metastases.

Metastases preferentially affect areas rich in hematopoietic marrow. In lower limbs, the femur is more likely than the tibia to be affected by secondary cancer localization. Involvement of the tibia is uncommon [58], [59]. Most lesion topography is intramedullary.

Metastatic bone disease is often asymptomatic. It may also be revealed by pain, a pathological fracture, a change in general condition or the discovery of biological abnormalities, particularly in phospho-calcium metabolism.

**Fig. 12:**
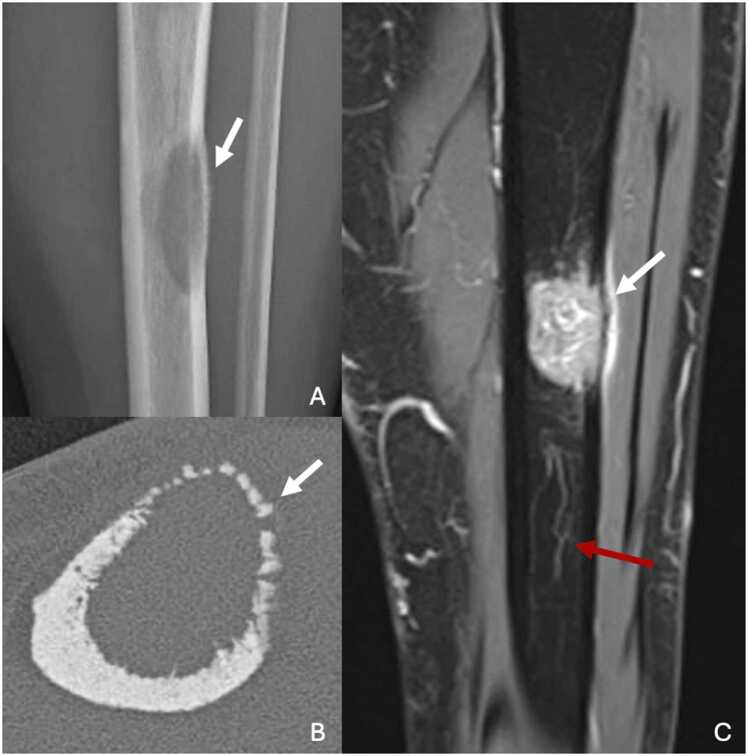
Corticomedullar metastase of the middle third of the left tibia in a 74-year-old woman with a known endometrial neoplasia. A. Face radiography of the left tibia showing a radiolucent oval-shaped corticomedullar lesion with discreet periosteal reaction (white arrow). B. UHR CT image in the axial plane highlighting the periosteal reaction with moth-eaten appearance of the anterior cortex (white arrow). C. T1-weighted fat-suppressed and contrast enhanced MR image in the sagittal plane showing periosteal enhancement adjacent to the metastase, mainly centromedullar. Notice the peritumoral vascularization (red arrow).

On imaging, metastases vary in appearance, but around 75 % of lesions are osteolytic [60]. Peripheral sclerosis is rarely present. Periosteal reaction is often weak (Fig. 12).

Detection of osteolytic metastases is not always easy on standard radiographs, especially if the lesions are less than one cm in size and involve less than 50 % of cortical thickness. CT scans allow better visualization of these secondary lesions, as well as better assessment of cortical damage. The risk of additional pathological fracture can be calculated with the Mirels score [61].

Osteocondensing metastases are rarer and are generally the site of disseminated breast or prostate cancer. Their boundaries are often blurred and not spiculated, unlike enostoses. Mixed lytic and condensing lesions are also possible.

On MRI, osteolytic lesions appear hypointense on T1-weighted images and hyperintense on T2, while osteocondensing lesions are hypointense on both sequences. Many secondary lesions display a characteristic "target sign", with peripheral T2 hyperintensity [62]. Metastases show contrast outside necrotic portions.

Bone scintigraphy has excellent sensitivity, detecting over 90 % of secondary bone lesions. However, specificity is low, as many bone lesions are hyperfixed. ^18^FDG PET scan is of interest for metastases of cancers with high metabolic activity, such as breast, lung, colon and lymphoma. Bone metastases of some cancers, such as prostate, are not hypermetabolic.

## 5 Conclusion

In this first part of our two-part review, we have provided a detailed examination of dysplastic and diaphyseal lesions of the tibia, offering a systematic approach to diagnosis through imaging and clinical context. This article presents diagnostic algorithms and imaging features designed to facilitate the differentiation between tumoral, non-tumoral and dysplastic lesions.

In the second part of this review, we will focus on metaphyseal and epiphyseal lesions of the tibia, continuing to build on the diagnostic frameworks and imaging findings discussed here.

## Declaration of Generative AI and AI-assisted technologies in the writing process

During the preparation of this work the authors used ChatGPT in order to improve language and readability. After using this tool/service, the authors reviewed and edited the content as needed and take full responsibility for the content of the publication.

## Funding statement

This work did not receive any specific grant from funding agencies in the public, commercial, or not-for-profit sectors.

## Declaration of Competing Interest

The authors declare that they have no known competing financial interests or personal relationships that could have appeared to influence the work reported in this paper.
